# Practice-level mechanisms contributing to successful implementation of a UTI antibiotic stewardship intervention: a qualitative study in Dutch general practices

**DOI:** 10.1186/s12875-026-03233-5

**Published:** 2026-03-16

**Authors:** Lisa Powaga, Esther de Groot, Esther A. R. Hartman, Tamara N. Platteel, Roderick P. Venekamp, Cees M. P. M. Hertogh, Nienke Bleijenberg, Dorien L. M. Zwart, Alma C. van de Pol

**Affiliations:** 1 Department of General Practice & Nursing Science, Julius Center for Health Sciences and Primary Care, University Medical Center Utrecht, Utrecht University, Utrecht, the Netherlands; 2 Department of Medicine for Older People, Amsterdam UMC, Vrije Universiteit Amsterdam, De Boelelaan 1117, Amsterdam, the Netherlands; 3 Aging & Later Life, Amsterdam Public Health Research Institute, Amsterdam, the Netherlands

**Keywords:** Antibiotic stewardship intervention, Older adults, Implementation, Qualitative research, Healthcare professionals, Urinary tract infections, Practice-level mechanisms, General practice

## Abstract

**Background:**

A recent multi-country cluster randomised trial (ImpresU) found that implementation of an antibiotic stewardship intervention (ASI) safely reduced antibiotic prescribing for suspected urinary tract infections (UTI) in frail older adults. However, it remains to be elucidated how the ASI achieved organisational change at practice-level. Therefore, the aim in this study, was to unravel practice-level mechanisms contributing to successful implementation of the ImpresU UTI ASI in Dutch general practices.

**Methods:**

A qualitative interview study with healthcare professionals from general practices and the older adult care organisations was performed. These general practices and older adult care organisations were part of all six Dutch intervention clusters in the ImpresU trial, where the UTI ASI has led to fewer antibiotic prescribing. Semi-structured interviews were performed with 15 health care professionals between November 2023 and February 2024. Interviews were transcribed verbatim and analysed using thematic analysis.

**Results:**

We identified four practice-level mechanisms contributing to successful UTI ASI implementation: (1) scheduling a dedicated moment for interprofessional discussions about suspected UTIs, (2) making step-by-step work instructions visually available for all health care professionals involved, (3) structured steps and nudging to facilitate adoption of new practices and (4) enhancing motivation by a team champion. All of the mechanisms together seem to have led to a more adequate UTI care pathway in the general practices, through which they could reduce antibiotic prescribing.

**Conclusion:**

We identified four key practice-level mechanisms through which Dutch general practices implemented an ASI to improve their UTI care pathway for frail older adults. In-depth insight into how implementation works through these mechanisms can inform strategies for broader UTI ASI implementation in diverse general practice settings.

**Supplementary Information:**

The online version contains supplementary material available at 10.1186/s12875-026-03233-5.

## Background

Diagnosing urinary tract infections (UTIs) in frail older adults in general practice is challenging. Non-specific symptoms such as smelly or cloudy urine, or behavioural changes are often mistaken for symptoms of UTIs [1, 2]. Additionally, the prevalence of asymptomatic bacteriuria is high in this population, hence if a urine sample is tested in patients with non-specific symptoms, it is often positive [3]. Together, these challenges lead to the overdiagnosis of UTIs in older adults in general practice and, consequently, overuse of antibiotics [2, 4]. Antibiotic overuse not only increases the likelihood of side effects for the individual but also poses a risk of antibiotic resistance for both the individual and the community [1]. Therefore, there is a need to understand how to improve antibiotic prescribing practices for older adults.

An antibiotic stewardship interventions (ASI) is a targeted action designed to promote the appropriate use of antibiotics and is key in optimising antibiotic prescribing for UTIs [5]. Numerous ASIs consisting of components such as clinician education and clinical decision support have shown promise in improving general practitioners’ (GPs) antibiotic prescribing routines, with multifaceted interventions being more effective than those consisting of a single component [6, 7]. However, little is known on how these multifaceted ASIs successfully changed prescribing routines at the level of the practice organisation. In the Netherlands, the organisation of general practice care for older adults suspected of having a UTI is complex, as it requires the actions of multiple healthcare professionals. For example, for appropriate symptom recognition and urine testing, nursing staff at an older adult care organisation, general practice assistants and general practitioners need to collaborate. To reduce antibiotic prescribing, change at the organisational level is warranted. To understand how such a change can be achieved, one will need to look beyond the actions of individuals alone and focus on the practice-organisation level.

A recent multi-country cluster randomised trial, ImpresU, found that implementing a UTI ASI safely reduced antibiotic prescribing for suspected UTIs in frail older adults compared with usual care [8]. The core of the ASI consisted of a new way of working: a decision-tool to promote appropriate antibiotic use, previously developed using a Delphi procedure [9]. The ASI was implemented using a participatory-action-research (PAR) approach with sessions for education and evaluation (Table 1) [1]. We found that in Dutch general practices, health care professionals adapted their local organisation of care with the ASI [10].

**Table 1:** Overview of the design and implementation of the UTI ASI

**Antibiotic stewardship intervention**
1) Decision tool – guiding appropriate antibiotic use based on the presence of specific UTI symptoms
2) Educational toolbox – includes materials such as pocket cards, posters, informational brochures
**Implementation strategy using participatory-action-research approach to actively involve all healthcare professionals**
1) Educational sessions – healthcare professionals learn how to recognize a UTI using the decision tool, reflect, and plan implementation
2) Evaluation sessions – healthcare professionals reflect and plan further implementation

In this study, we explored how the UTI ASI led to change at the level of the practice-organisation in the Dutch general practices that were part of the intervention arm of the ImpresU trial. Our studies’ aim was to unravel practice-level mechanisms contributing to successful implementation of the ImpresU UTI ASI in Dutch general practices, through an in-depth exploration with healthcare professionals from Dutch general practices and the older adult care organisations where the UTI ASI was implemented.

## Method

### Study design and setting

We conducted a qualitative study using semi-structured interviews with healthcare professionals from general practices and the older adult care organisations (residential care homes and home care organisations) where general practices provide medical care. At least one participating practice from each of the six Dutch intervention clusters of the ImpresU trial was included [8]. In the Netherlands, GPs serve as gatekeepers to specialist care, and interprofessional collaboration is a key focus within primary care. In most Dutch general practices, an interprofessional team consisting of GPs, general practice assistants (GP assistants), general practice nurses (GP nurses) and other professionals, work together to provide comprehensive care to their patients. This study was conducted and reported according to the Standards for Reporting Qualitative Research framework [11].

### Participant selection and recruitment

We purposefully selected healthcare professionals from all Dutch general practices and the older adult care organisations that were part of the intervention arm of the ImpresU trial. This sampling strategy aimed at identifying those individuals who could provide rich, in-depth insights relevant to the research question [12, 13]. We interviewed general practitioners, general practice assistants, general practice nurses, and nurses from older adult care organisations. A researcher (EH) approached 20 potential participants by e-mail. These individuals were all local study coordinators from each of the six intervention clusters, representing the relevant healthcare professional groups. Interested participants were contacted by telephone and received a study information leaflet by e-mail. Participants had to be able and willing to provide verbal informed consent prior to the interview.

### Data collection and management

We developed a semi-structured interview guide (Additional file 1), based on literature and the researchers’ experience [8, 14]. The interview guide was pilot-tested with two GPs, after which minor changes were made to the interview guide. Data from the pilot-interviews were not included in the analysis. During the interviews, we asked participants about their experiences with the multiple components that were part of the ASI, as well as their experiences with the PAR approach. Furthermore, we asked them if and how changes were made in the UTI care pathway of their practice.

The first interview was conducted by an experienced researcher (EH) and a junior researcher (LP). Because EH was involved in the conduct of the ImpresU trial and had previous contact with the healthcare professionals, all interviews following participant one were conducted by a single researcher (LP), who had no prior relationship with the participants and no previous involvement in the ImpresU trial. This approach was chosen to provide a more neutral perspective during data collection. Interviews took place between November 2023 and February 2024, in person or via a video call (Microsoft Teams) based on participants’ preferences. Interviews were audio-recorded and transcribed verbatim by an independent professional transcription agency or the researcher. Transcripts and demographic participant data were pseudonymized and stored in a research folder at the University Medical Centre Utrecht, which was only accessible to the authorized researchers.

### Data analysis

Data analysis was done using thematic analysis [15]. The first step in the data analysis was conducted collaboratively by two researchers (AP and LP). Initially, they both read all transcripts to familiarise themselves with the data and discussed notable aspects. Subsequently, each researcher independently coded half of the transcripts and reviewed the codes assigned by the other researcher. Any discrepancies were resolved through discussion to reach consensus, involving a third researcher if needed (EG). The coded transcripts were then imported into Nvivo V.20. Codes were organized to identify initial themes, and these were further discussed with the wider research team. This team consisted of researchers with different expertise, allowing for critical reflection on the themes and reduced influence of individual perspectives. Subsequently, framework matrices were developed to systemically map initial themes both across the different clusters (represented on the x-axis) and the different type of healthcare professionals (represented on the y-axis) (AP, LP and EG). These matrices allowed identification of initial themes based on their relevance rather than on frequency of occurrence within highly sampled clusters. The research team analysed these matrices iteratively, aiming to identify overarching themes describing mechanisms at the general practice level (from now on called: mechanisms). Finally, we developed a model showing how these mechanisms impacted the UTI care pathway in general practices.

### Ethical considerations

This study does not fall under the scope of the Dutch Medical Research Involving Human Subjects Act (WMO). It therefore does not require approval from an accredited medical ethics committee in the Netherlands [16]. However, in the UMC Utrecht, an independent quality check has been carried out to ensure compliance with legislation and regulations (regarding Informed Consent procedure, data management, privacy aspects and legal aspects).

## Results

In total, fifteen interviews were conducted with four GPs, two GPs assistants, three GP nurses, and six nurses from older adult care organisations. An overview of characteristics from these healthcare professionals is summarised in Table 2.

**Table 2:** Characteristics of healthcare professionals from Dutch general practices and the older adult care organisations. These practices were part of all six Dutch intervention clusters of the ImpresU trial

Function	Gender (F/M)	Practice type
**Cluster 1**		
*A general practice (~ 2 GP’s) in a rural village providing medical care in an adjacent residential care home (around 60 patients). The GP nurse made weekly visits to the RCH.*		
GP	M	General practice
GP nurse	F	General practice
Nurse	F	Residential Care Home
Nurse	F	Residential Care Home
Nurse	F	Residential Care Home
**Cluster 2**		
*Three urban general practices*,* together providing medical care in a small-scale living facility for older people with dementia (around 20 patients).*		
GP	F	General practice 1
GP assistant	F	General practice 1
GP nurse	M	General practice 2
Nurse	F	Residential Care Home
**Cluster 3**		
*A general practice (~ 6 GP’s) providing medical care in a private residential care home (around 20 patients) and with two home care organisations*,* all in the same village.*		
GP	F	General practice
GP nurse	F	General practice
**Cluster 4**		
*A general practice (~ 6 GPs) with an older adult care organisation providing medical care in part of an adjacent residential care home and nearby apartments with home care*,* all in the same village. The care organisation did not participate in the sessions for education and evaluation.*		
GP	F	General practice
GP assistant	F	General practice
**Cluster 5**		
*A general practice (~ 3 GPs) in a small town providing medical care in a residential care home (around 15 patients)*,* and a home care organisation (around 25 patients). The general practice did not participate in the sessions for education and evaluation.*		
Nurse	F	Home care organisation
**Cluster 6**		
*Ten collaborating general practices (~ 2 GPs in each practice) in the same village*,* providing medical care in a large residential care home (around 80 patients).*		
Nurse	F	Residential Care Home

*GP* general practitioner, *M* male, *F* female

### Practice-level mechanisms contributing to successful UTI ASI implementation

Four practice-level mechanisms (themes) were found to contribute to successful UTI ASI implementation in the general practices.

#### Scheduling a dedicated moment for interprofessional discussions about suspected UTI’s

In the general practice, GPs and GP assistants agreed on having daily scheduled moments to discuss urine test results and patient symptoms with each other.

*Yes*,* that is also something that perhaps through ImpresU*,* we agreed on back then – because before we also said it*,* but it happened less often – but we also said “we really need to discuss those urine tests at the computer.’’ (GP 4)*.

Having interprofessional discussions may help to reinforce shared understanding about how UTI symptoms should be interpreted and managed.

*So if questions came from colleagues like “shouldn’t we check the urine?” The answer was yes okay*,* but is there more going on with this patient? Yes or no? No? Okay*,* well then I don’t think it’s necessary. (nurse 1).*

Conversely, not having these scheduled moments during the day brought some challenges for practices.

*Those urine tests come to us intermittently. So there are no fixed times*,* and I can be called during my consult with “Can I discuss a urinary tract infection for a moment?” And then a little story is rushed through and sometimes*,* well*,* I think based on the story*,* well*,* yes*,* that’s correct*,* um*,* go ahead with that antibiotic. Sometimes I do take a quick look at the file. But it happens in between while I’m busy with all sorts of other things. (GP 2).*

#### Making work instructions visually available for healthcare professionals

Making the same work instructions visually available for all employees within their practices appeared to support healthcare professionals in which actions to take. For example, multiple general practices incorporated structured urine forms to systematically identify and document patients’ symptoms (for example: dysuria, pollakiuria, flank pain, fever…). The primary purpose of these urine forms was to create a comprehensive and structured overview for GP assistants of symptoms to be documented. Practices that already used a urine form, further enhanced them to be more suitable for frail older patients. Additionally, using these forms facilitated the discussion about suspected UTI’s during the scheduled moments between GP and GP nurses, as previously described.

*So that the questionnaire [urine form] is completed. What are the symptoms? Are there symptoms indicating a urinary tract infection? Do people have abdominal pain? Frequent urination? And so on. Is there a fever? All measurements are taken and recorded. (GP nurse 1).*

*Yes*,* that [urine form] was already in place*,* but we have refined it a bit. We particularly emphasized that the form must be completed and used. (GP 3).*

The pocket cards from the toolbox showing the decision-tool also served as work instructions within practices. Pocket cards were used as a visual reminder in case healthcare professionals were uncertain about which actions to take with a patient.

*And I also think*,* if you have more doubts*,* then I’ll take this [pocket card]*,* because I know it’s in my bag and that it’s there. And it’s precisely those things that make you think*,* well*,* let’s take a moment to think extra*,* should we take another look? That’s how I see these kinds of decision-tools. (GP nurse 3).*

In older care organisations, posters with the decision-tool were placed in various locations to remind nurses of symptoms indicative of a UTI and the actions they should take. They were used by nurses during daily handover meetings, to raise awareness of the recommended actions by referring to the poster.

*During the handover I’m often present and it’s like ‘’Yes*,* we need to do a urine test because the patient is a bit confused.’’ And then we also refer to that decision tree and think ‘’Yes maybe it’s not really necessary yet*,* we can wait a bit longer.” (Nurse 6)*.

#### Structured steps and nudging to facilitate adoption of new practices

The adoption of new practices appeared to be facilitated through the incorporation of structured steps and nudges. A concrete example of such a structured step is that the GP automatically requested a completed urine form (filled in by nurses) along with each urine sample they wanted to deliver to the general practice.

*So we always have to*,* as per the agreement with the GP*,* we always have to submit this completed list along with the sample*,* so they know we have already conducted a broader examination. (nurse 2).*

By requiring that a completed urine form accompanies each urine sample, the general practice introduces a structured step for nurses to use urine forms and conduct a thorough assessment of patient symptoms before submitting samples.

Another example of a structured step was that in some older adult care organisations urine strips were put away and could only be used with a GP’s authorization.

*At a certain point*,* we just put the urine strips away. Like*,* ‘’these are only for emergencies and only if the GP says you can use them*,* then you can use them*,* otherwise not.’’ (nurse 3)*.

By placing the urine strips out of immediate reach and making sure that they could only be used with a GP’s authorization, the general practice promoted the nurses of the older adult care organisations to consider the necessity of using the strips.

An example of a nudge was placing pocket cards for nurses in front of a “UTI bin’’, holding all materials needed to start UTI-diagnostics, thereby facilitating the adoption of new practices among healthcare professionals.

*And we have a bin that contains those urine strips*,* the forms*,* and an instruction sheet [pocket card] on the front that explains when you can use the test strips for urine and what actions to take. (nurse 3).*

By adding the pocket card to the UTI bin and then placing it in one specific location, healthcare professionals were nudged to perform the appropriate actions at the moment they are at the box. They automatically, without even realizing it, used the work instruction due to the way it was attached to the UTI bin. 

#### Enhancing motivation by a team champion

Some teams were encouraged to adopt the new way of working by one specific health care professional in the team. This team member seemed to play a crucial role by proactively fostering an innovation culture within the organisation. Other team members mentioned the active engagement of this team champion promoted shared behavioural change. The constant motivation and involvement of the team champion played a crucial role in maintaining adherence to the correct procedures by others.

*She is very involved in all those processes*,* more so than the GPs themselves. She also guides her colleagues*,* so that’s… Yeah*,* she also makes sure that we adhere to those procedures*,* you know. (nurse 1).*

A team champion herself, she noticed how her enthusiasm for the new way of working motivated others to embrace it as well.

*Because they saw that I was very enthusiastic and working hard on it*,* you also bring people along from your team. And that’s the way that works best*,* when people make the choice themselves. (GP nurse 1).*

Controversially, some practices lacked a team champion, which was seen as a vulnerability.

*As a nurse assistant - who often knows the older people well because she has been there for so long - if she still does not take on that responsibility*,* I think that has been a limiting factor. (GP nurse 2).*

### Model visualizing how the four practice-level mechanisms impacted the UTI care pathway in general practices

**Fig. 1:**
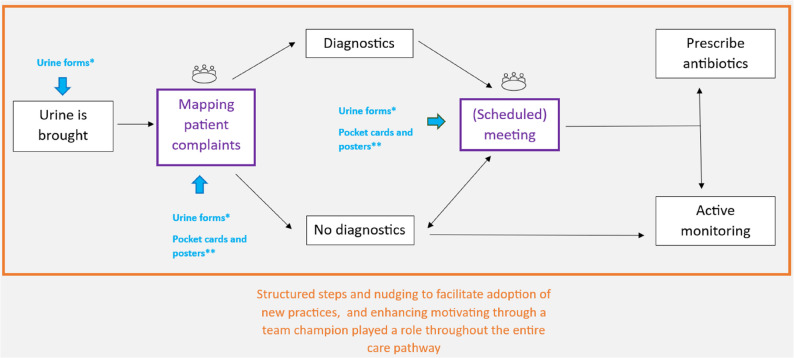
Model visualizing how the four practice-level mechanisms impacted the UTI care pathway in general practices. Blue: Making work instructions visually available for healthcare professionals. Purple: Scheduling a dedicated moment for interprofessional discussions about suspected UTI’s. In some general practices, often a meeting moment is held between the GP and GP assistant after urine diagnostics is performed, or when the GP assistant is not sure what decision should be made next. However, this moment can happen throughout the day and in between other tasks. Therefore we placed ‘’scheduled’’ between brackets, because it does not necessarily take place at a scheduled moment during the day. Orange: structured steps and nudging, and motivating through a team champion. * Urine forms (work instruction) were used to systematically identify and document patients symptoms. ** Pocket cards and posters with the decision-tool (work instruction) served as visual reminders of the recommended actions to take

All four practice-level mechanisms impacted the general practice UTI care pathway, helping in successful implementation of the ASI. Overall, the UTI care pathway seemed to become more adequate (see Fig. 1). When urine was brought, symptoms were systematically mapped using urine forms. Other work instructions such as pocket cards and posters were used to remind everyone in the general practice about which actions to take: (a) diagnostics or no diagnostics, and (b) prescribe antibiotics or active monitoring. The latter action was decided upon after scheduled moments. During this moment, the symptoms mapped, were discussed. Structured steps and nudging to facilitate adoption of new practices, and motivation through a team champion played a role throughout the entire UTI pathway.

## Discussion

The aim of our study was to unravel practice-level mechanisms contributing to successful implementation of the ImpresU UTI ASI in Dutch general practices. We identified four key mechanisms through which new ways of working and reductions in antibiotic prescribing may have been achieved.

### Comparison with previous research

The current qualitative study builds on our previous quantitative multi-country process evaluation, in which we evaluated the different components of the ASI implementation based on a questionnaire (surveys) with healthcare professionals and documentation of the implementation process [10]. By performing a more in-depth qualitative exploration with these healthcare professionals, we gained understanding in the mechanisms through which general practices implemented the intervention at their practice-level, achieving a reduction in antibiotic prescribing. For instance, the process evaluation found the motivation of health care professionals for the ImpresU trial to be an important facilitator for implementation [10]. Our qualitative interview approach allowed us to deepen our understanding on the role of these health care professionals at the level of the general practice.

A study of Hye-Ryun Kang et al. (2006), examining the importance of team member characteristics on team effectiveness, showed that team effectiveness is more influenced by cognitive than demographic similarities. Carraro et al. (2024) conducted a cross-sectional survey study on how shared mental models influence team level problem-solving and found that greater alignment of task- and team-related mental models enhances proactive problem-solving and coordinated team effort, improving team performance. Both studies support that shared understanding among team members enhances team effectiveness by improving coordination and collaboration [17, 18]. One of the main mechanisms was having scheduled moments for interprofessional discussions on how suspected UTIs should be managed. Together with the second key mechanism, using step-by-step work instructions, the healthcare professionals seemed to gain a better shared understanding of actions to take: (a) diagnostics yes or no, and (b) antibiotic prescription yes or no. Similarly, a qualitative comparative process evaluation of Røsstad et al. (2015) showed that creating engagement and commitment among professional groups, e.g. through discussing their intervention iteratively in team meetings, was a factor for successful implementation [19]. Having discussions appeared to aid in building a shared understanding among all healthcare professionals in the general practice.

The adoption of the new way of working was facilitated using structured steps and nudges. Nudging is a well-established strategy for influencing people’s choices by altering their decision-making environment and has been widely used in health services and public health [20, 21]. Raban et al. (2022) conducted a systematic review of nineteen studies evaluating nudge interventions to reduce unnecessary antibiotic prescribing in general practice. Overall, nudge interventions may be effective for improving antibiotic prescribing [21]. Our UTI ASI did not include a specific nudge, per se, yet we found that healthcare professionals were prompted by the UTI ASI to start nudges at their own initiative, which helped them to achieve the desired change.

Finally, having a dedicated team champion to achieve change within a team has been previously shown instrumental for successful implementation at a practice-level. A qualitative study of Davis et al. (2017), using focus groups and interviews with clinic and community-based users, showed that effective translation of intervention toolkits into practical actions requires support, such as a dedicated individual facilitating its adoption [22]. In a qualitative interview study, Kousgaard et al. (2022) found nurses to be team champions in ASI implementation by explaining and discussing the ASI to other staff [23]. Although identifying and preparing champions was not an explicit component of the ImpresU trial ASI, our analyses revealed that certain healthcare professionals adopted this role. They felt this fostered change, and this was also recognized by others as an important factor for success.

The model we developed illustrates how the four practice-level mechanisms influence each other and collectively impact the UTI care pathway in general practices (see Fig. 1). The mechanisms closely align with several constructs within the inner setting domain as outlined in the Consolidated Framework for Implementation Research (CFIR) framework (i.e. the practice-level). This includes structural characteristics, network and communication, and implementation climate (with subconstructs such as compatibility and leadership engagement) [24]. Based on barriers identified within the CFIR constructs, the following implementation strategies - recommended by the Expert Recommendations for Implementing Change (ERIC) - were components of the ASI in the ImpresU trial: organising clinician implementation team meetings, conducting local consensus discussions, promoting adaptability, and identifying and preparing champions [25]. These seem to have worked through the four mechanisms, together contributing to a new way of working. Although our analysis focused on identifying themes describing mechanisms at the general practice level, we have the impression that the four mechanisms may have also played a role at the level of older adult organisation, given the close collaboration between both. This collaboration could have impacted the UTI care pathway of the general practices. However, our focus was not on the setting of healthcare organisations.

### Strengths and limitations

A major strength from this study was the identification of mechanisms at the practice-level and its focus on the broader organisational context. This level has been underexplored in the implementation of ASIs, even though it is well-recognized that change in organisation does not solely rely on actions at the level of single individuals. Another strength of this study was that it integrates different perspectives from all healthcare professionals within the general practices. By capturing the views of all these healthcare professionals, the study offers a nuanced understanding of how collaborative practices influence antibiotic prescribing decisions. Interviews conducted via video call (Microsoft Teams) facilitated participation of healthcare professionals across different regions and clusters.

A limitation of this study was that not all types of healthcare professionals in each cluster responded to our invitation, and that this might be related to the enthusiasm for the intervention in a cluster. Interviewing more healthcare professionals in an intervention practice could have provided additional insights. However, the diverse group of respondents allowed us to capture the perspectives from each type of healthcare professional and ensured representation from at least one practice within each Dutch intervention cluster.

### Implications for practice

Our study highlights the importance of adopting an approach targeting the practice-level to implement changes in the UTI care pathway of general practices, rather than focusing on individual efforts. Our study suggests that successful implementation requires a combination of mechanisms. The identification and in-depth understanding of these mechanisms will allow each general practice organisation to explore how these different mechanisms will work in their specific local context. Furthermore, practices could make small adjustments in the general practice to efficiently adopt the new UTI care pathway. Amongst others, one could consider putting away urine sticks in older adult care organisations to make sure they cannot be used without discussing symptoms first, and/or automatically hand out a urine form with each urine container. To keep the team motivated and foster change, they can identify a colleague who want to serve as team champion (Table 3). Within the general practice, one can reflect on the current practices, discuss the goals, and collectively determine the steps needed as a team to make those goals a reality.

**Table 3:** Recommendations for change in the general practice, based on the identified mechanisms

**Schedule a dedicated UTI moment**
Choose a scheduled moment (for example at 11 AM) for the GP assistant and GP to discuss urine test results and patient symptoms, instead of having these moments in between other tasks
**Make work instructions visually available**
Keep pocket-cards or posters available for health care professionals to easily find the work instructions
**Adopt new practices through structured steps and nudging**
Put away urine sticks in older adult care organisations to make sure they cannot be used without discussing symptoms first
Attach a pocket-card with a work instruction to the UTI bin (holding all materials needed to start UTI-diagnostics) so that health care professionals use it automatically, almost without realising
**Enhance motivation by a team champion**
During a team meeting, identify a colleague who is willing to serve as team champion regarding the UTI care pathway

### Implication for future research

Although the ASI from the ImpresU trial was found to be effective, nationwide implementation of the intervention poses a challenge. During the trial, researchers visited each general practice, a process that is not feasible on a national level. Therefore, all relevant stakeholders are currently co-designing a UTI ASI strategy for broader implementation, maintaining the components that allow the mechanisms to work effectively. For example, we will not only rely on an online learning to enhance individual knowledge (an implementation strategy that is easily scalable), since this is insufficient to drive change at the practice-level. Building on the identified mechanisms, we plan to include a team champion as part of the broader UTI ASI strategy and evaluate its effect on implementation. The practice-level mechanisms identified in the current study will allow other organisations and researchers to design and evaluate ASI strategies that fit within their unique context.

##  Conclusion

We found four practice-level mechanisms through which Dutch general practices implemented an ASI to improve their UTI care pathway for frail older adults, when conducting a multi-country cluster randomised trial. This will help the development of future strategies for broader implementation of UTI ASIs in Dutch general practices. The insight gained in these mechanisms allows health care professionals, in other countries and with diverse organisations of UTI care pathways, to design and evaluate ASI strategies that fit within their unique context.

## Supplementary Information

Supplementary Material 1.

## Data Availability

The data can be made available for researchers whose proposed use of the data has been approved at request of the corresponding author, with a signed data access agreement.

## Abbreviations

ASI: Antibiotic Stewardship Intervention
CFIR: Consolidated Framework for Implementation Research
ERIC: Expert Recommendations for Implementing Change
GP assistants: General Practice Assistants
GP nurses: General Practice Nurses
GPs: General Practitioners
PAR: Participatory–Action–Research
UTI: Urinary Tract Infections
