# Supplementary material for: Practice-level mechanisms contributing to successful implementation of a UTI antibiotic stewardship intervention: a qualitative study in Dutch general practices
Source: BMC Prim Care. 2026 Mar 16;27:193. doi: 10.1186/s12875-026-03233-5 (PMC13181932; doi:10.1186/s12875-026-03233-5)
Supplement: Supplementary file 1 — Supplementary Material 1. [file 12875_2026_3233_MOESM1_ESM.pdf]

Additional file 1– Interview guide

Topic	Main questions	Follow-up questions
General information, personal background and profession	- You work as a [position] in a general practitioner's office/healthcare organisation, is that correct?<br>- Could you briefly tell me about your practice/organisation and how long you have been working there?<br>- What is your age?	
Approach to UTIs	As you know, I am interested in urinary tract infections among frail older adults in this research.<br><br>- Can you tell me about a recent case in which you were involved regarding this?<br><br>- Do you have another example of a case where antibiotics were/were not prescribed?	- How was this handled at that time?<br>- What was your role in how this was handled?<br>- What was the role of others? Do you have insight into that?<br>- To what extent do you notice a change?<br>- How did you feel it went?<br>- What can be improved?<br>- Is it going well or not?
Role decision tool and toolbox	In the intervention, the decision tool was central. Its aim was to facilitate the decision-making process for prescribing antibiotics.	

	- What do you still know about this?<br><br><br><br><br>- Do you feel it has brought a change?<br><br><br>Additionally, a lot of educational materials were provided such as E-learning, pocket cards, posters...<br><br>- What do you still know about this?<br><br><br><br><br><br>- Do you feel it has brought a change?	- How was this organized in your setting?<br>- Do you have an example of this?<br>- What did you start doing differently?<br>- What did colleagues start doing differently?<br><br>- How do you notice this change?<br><br><br><br><br><br>- How was this organized in your setting?<br>- Do you have an example of this?<br>- What did you start doing differently?<br>- What did colleagues start doing differently?<br><br>- How do you notice this change?
Role participatory action research	Training sessions were also organized for all caregivers involved…<br><br>- Can you tell me, if you attended, how you experienced the training?	 <br><br>- Why was this useful or not?

	In addition, a multidisciplinary meeting was also organized. This was a short meeting together with general practitioners and nurses to evaluate how things are going…<br><br>- Can you tell me, if you attended, how you experienced the multidisciplinary meeting?	- Why was this useful or not?<br>- What was the effect of repetition?
Role collaboration	What do you think is the role of collaboration/teamwork in the diagnosis and treatment process of UTI in frail older adults?	- The role within one's own practice/care organisation?<br>- The role between practice and healthcare organisation?<br>- The role of collaboration with patients and their families?
End	Review interview guide to ensure all questions have been asked.<br><br>I don't have any further questions. It was a pleasant conversation, and I've learned a lot from you. Are there any other topics related to urinary tract infections in frail older adults that you would like to discuss before we conclude the interview?<br><br>Thank you for your participation.	
